# Supplementary material for: EP4-induced mitochondrial localization and cell migration mediated by CALML6 in human oral squamous cell carcinoma
Source: Commun Biol. 2024 May 14;7:567. doi: 10.1038/s42003-024-06231-4 (PMC11093972; doi:10.1038/s42003-024-06231-4)
Supplement: Supplementary file 2 — Supplementary Information [file 42003_2024_6231_MOESM2_ESM.pdf]

Supplementary information for

EP4-Induced Mitochondrial Localization and Cell Migration Mediated by CALML6 in Human Oral Squamous Cell Carcinoma

Soichiro Ishikawa[1,2,†], *Masanari Umemura[1,†], Rina Nakakaji[1,2], Akane Nagasako[1], Kagemichi Nagao[1], Yuto Mizuno[1], Kei Sugiura[2], Mitomu Kioi[2], Kenji Mitsudo[2], Yoshihiro Ishikawa[1]

[1]Cardiovascular Research Institute, Yokohama City University Graduate School of Medicine, Yokohama, Japan

[2]Department of Oral and Maxillofacial Surgery, Yokohama City University Graduate School of Medicine, Yokohama, Japan

* Corresponding authors

[†] These authors contributed equally: Soichiro Ishikawa, Masanari Umemura

Email:

umemurma@yokohama-cu.ac.jp (MU)

Yokohama City University School of Medicine, 3-9

Fukuura, Kanazawa-ku, Yokohama, Japan.

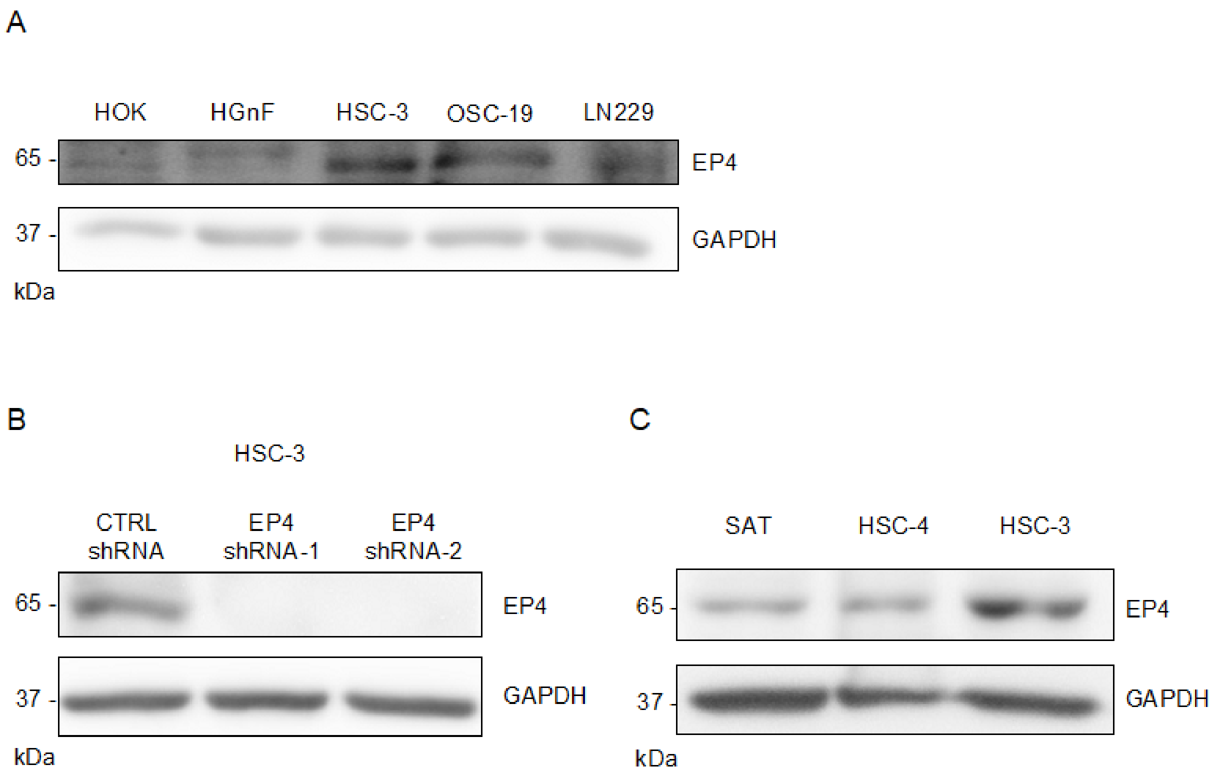

Supplementary Figure 1. The protein expression of EP4 in cancer cell lines was higher than that in normal cell lines.

A, Representative western blot (W.B.) analysis images showing EP4 protein levels in human cancer cell lines and normal cell lines (n=1).

B, Western blot (WB) analyses demonstrated a significant reduction in EP4 protein levels in HSC-3 cells treated with either EP4 shRNA-1 or EP4 shRNA-2 lentivirus, compared to HSC-3 cells treated with scramble shRNA lentivirus (n=1).

C, Representative western blot (WB) analysis images depicting EP4 protein levels in human oral cancer cell lines with varying metastatic potentials (n=1).

A

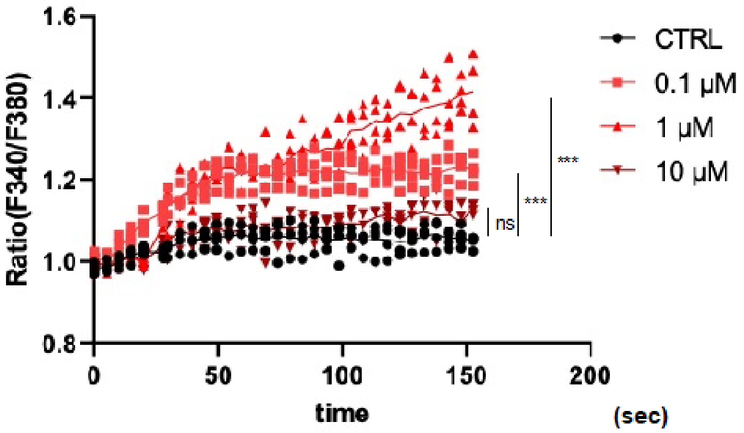

B

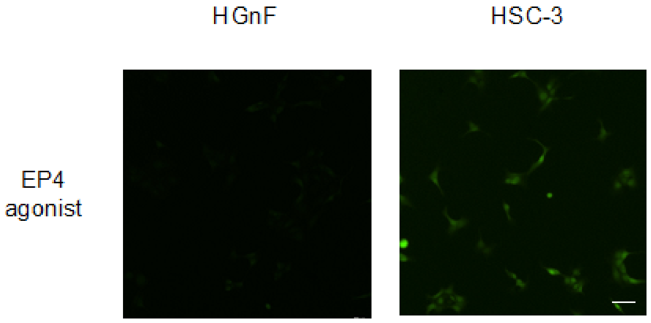

Supplemental Figure 2. EP4 activation increased Ca^{2+} levels in OSCC Cells but Not in HGnF Cells. **A,** Treatment with an EP4 agonist resulted in a significant increase in intracellular Ca^{2+} in HSC-3 cells, with the greatest increase observed at a concentration of 1 μM (One-way ANOVA, Tukey's multiple comparisons test; ***$p < 0.001$). These findings are based on four independent experiments ($n = 4$).

B, Fluorescence imaging revealed a significant increase in intracellular Ca^{2+} in HSC-3 cells following EP4 agonist treatment, in contrast to the negligible effect in HGnF cells (n=1).

Scale bars represent 100μm.

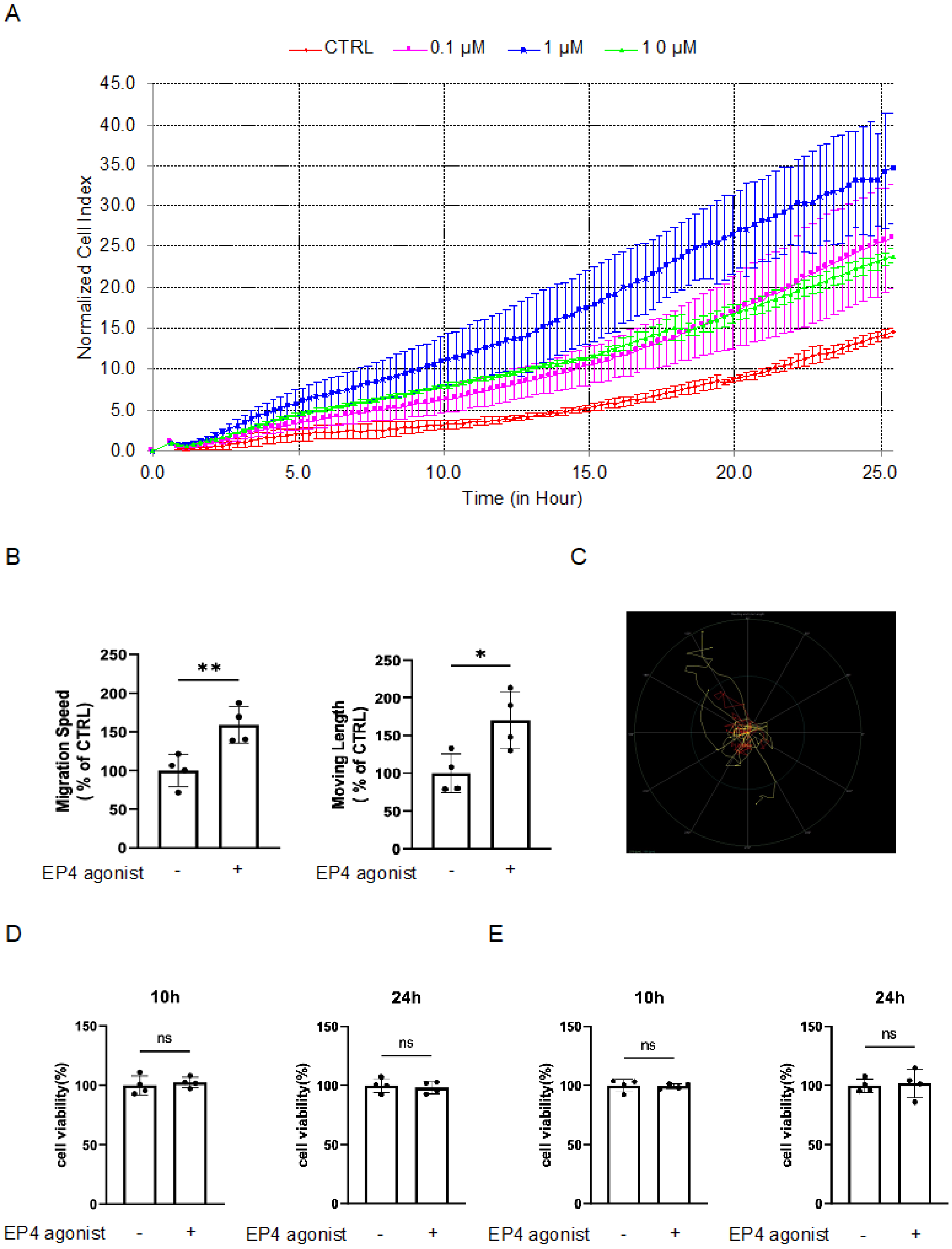
A
CTRL
0.1 μM
1 μM
1 0 μM
Normalized Cell Index
45.0
40.0
35.0
30.0
25.0
20.0
15.0
10.0
5.0
0.0
0.0
5.0
10.0
15.0
20.0
25.0
Time (in Hour)
B
Migration Speed (% of CTRL)
250
200
150
100
50
0
**
EP4 agonist - +
Moving Length (% of CTRL)
250
200
150
100
50
0
*
EP4 agonist - +
C
D
10h
cell viability(%)
150
100
50
0
ns
EP4 agonist - +
24h
cell viability(%)
150
100
50
0
ns
EP4 agonist - +
E
10h
cell viability(%)
150
100
50
0
ns
EP4 agonist - +
24h
cell viability(%)
150
100
50
0
ns
EP4 agonist - +

Supplemental Figure 3. Examination of migration speed and directionality in HSC-3 cells following EP4 agonist stimulation

A, The EP4 agonist enhanced cell migration in HSC-3 cells, as demonstrated by the xCELLigence Real-Time Cellular Analysis system. Treatment with the EP4 agonist resulted in a significant increase in cell migration, with 1 μM showing the most pronounced effect (*n*=3).

B, In the cell tracking migration assay, the EP4 agonist increased the speed and range of migration in HSC-3 cells, although the results were not statistically significant (unpaired t-test; ns, not significant; *n*=4). More than 10 cells were analyzed per field of view.

C, Polar plot graphs representing the migration of HSC-3 cells in the presence of the EP4 agonist. Each line corresponds to a single migrating cell. Red lines represent the control group, while yellow lines indicate the EP4 agonist-treated group. These plots suggest that directionality was enhanced by the EP4 agonist (*n*=10).

D, Cell proliferation of the human gingival fibroblasts was not changed by EP4 agonist after 10 or 24 hours (unpaired *t* test; ns; not significant, *n*=4).

E, Cell proliferation of the oral cancer cell line was not changed by EP4 agonist after 10 or 24 hours (unpaired *t* test; ns; not significant, *n*=4).

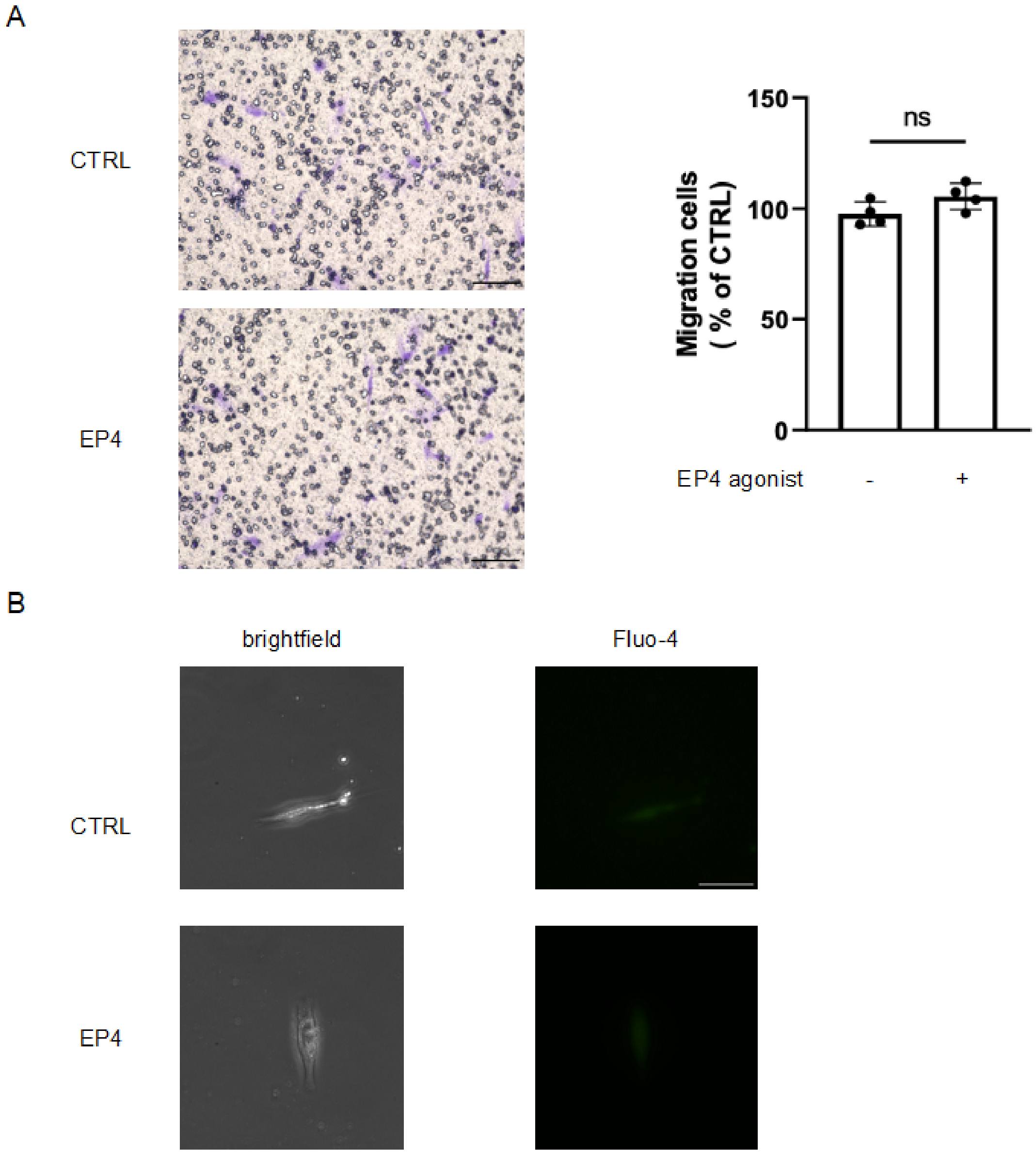

Supplemental Figure 4. Lack of effect of EP4 agonist on Ca^{2+} increase and cell migration in human oral keratinocytes (HOK).

A, Transwell migration assays demonstrated that the EP4 agonist did not enhance migration in HOK cells (unpaired *t*-test; ns, not significant; n=4). Scale bars represent 100µm.

B, Fluorescence imaging of intracellular Ca^{2+} indicated that the EP4 agonist did not induce an increase in Ca^{2+} levels in HOK cells (n=1). Scale bars represent 50µm.

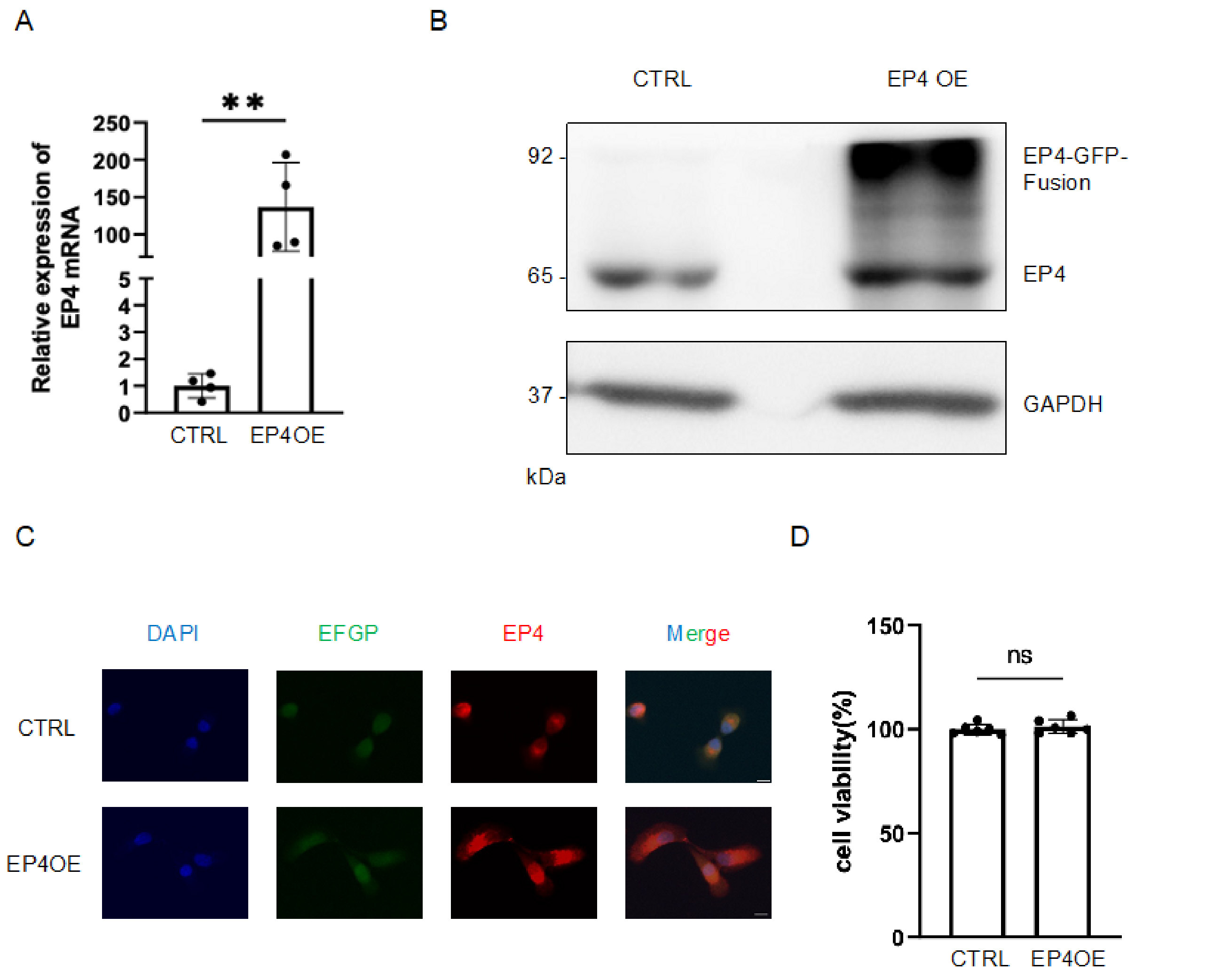

Supplemental Figure 5. EP4 overexpression did not change the proliferation of OSCC cells.

A, We evaluated the overexpression efficiency of EP4 using quantitative PCR. Compared to HSC-3 cells that had been introduced to the control lentivirus, HSC-3 cells that overexpressed EP4 exhibited much higher mRNA expression levels of EP4 (n=4).

B, We evaluated the overexpression efficiency of EP4 using WB. Compared to HSC-3 cells that had been introduced to the control lentivirus, HSC-3 cells that overexpressed EP4 exhibited much higher protein expression levels of EP4 (n=1).

C, Overexpression of EP4 in HSC-3 cells was verified through immunofluorescence staining. The left panel displays DAPI staining (blue) of both control and EP4-overexpressed HSC-3 cells. The

second image from the left shows EGFP (green), while the third image depicts EP4 (red). The right panel presents triple staining with DAPI (blue), EGFP (green), and EP4 (red). Overexpression of EP4 led to a minor increase in fluorescence intensity without noticeable changes in localization. More than 50 cells were analyzed per field of view. Scale bars represent 10μm.

D, The XTT assay showed that EP4 overexpression did not change cell proliferation (unpaired *t* test; ns; not significant at 24 h (n=6)).

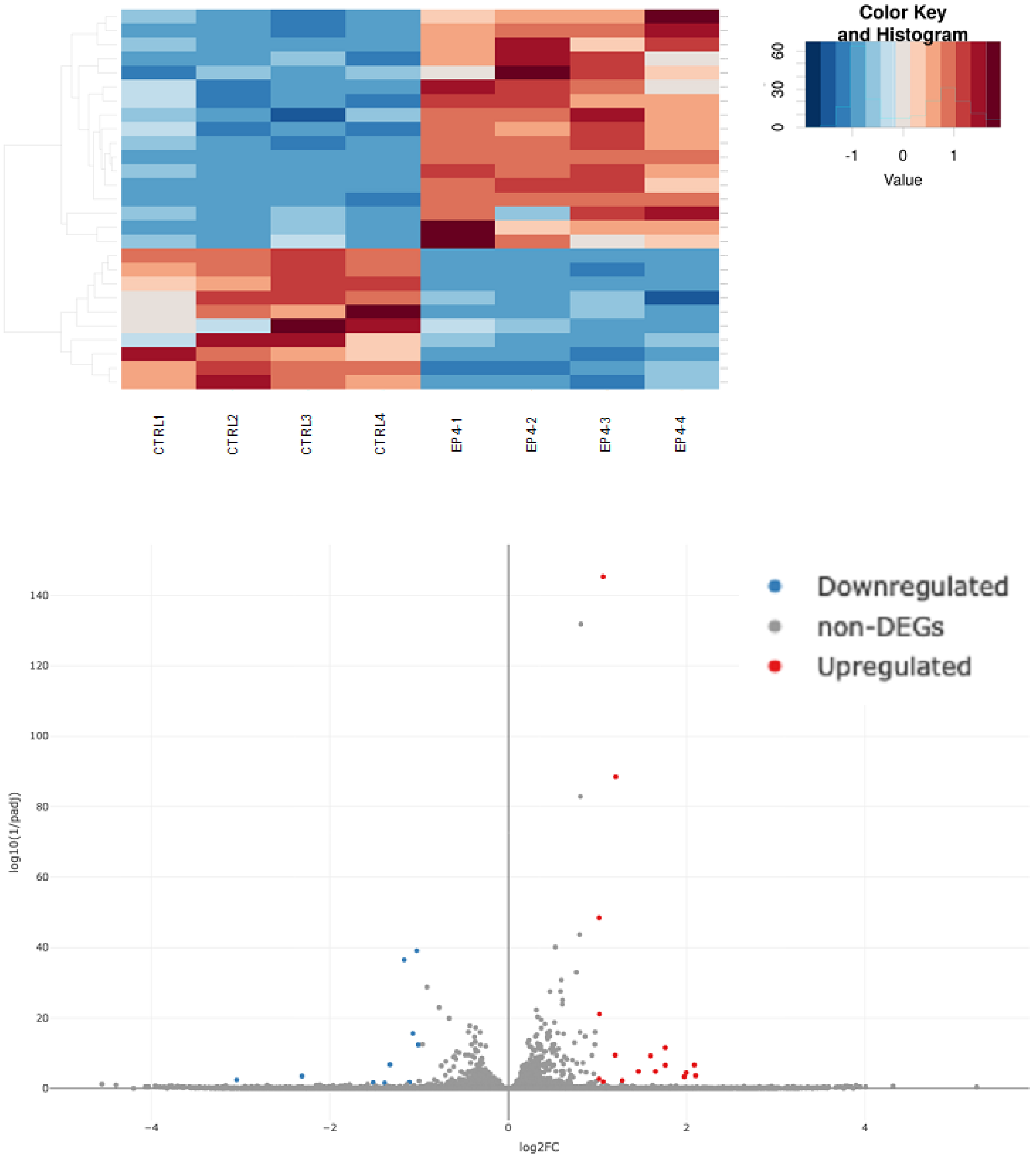

Supplementary Figure 6. Differential gene expression in oral cancer cells following treatment with EP4 agonist.

RNA-seq analysis was performed on HSC-3 cells treated with and without the EP4 agonist (n=4). The treatment resulted in the upregulation or downregulation of several genes. The figure includes a heatmap (*upper*) and a volcano plot (*lower*) illustrating these changes. Detailed information is available in the Source Data.

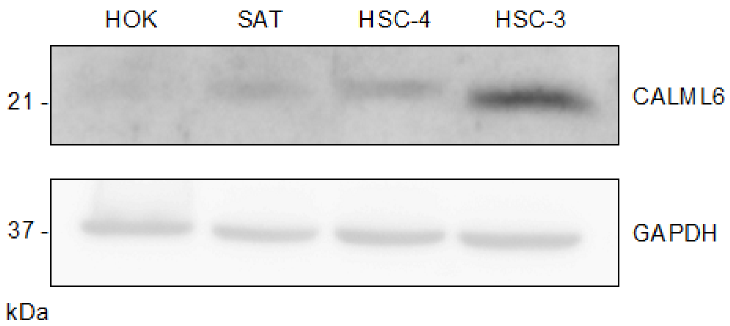

Supplemental Figure 7. Elevated CALML6 protein expression in oral cancer cell lines with high metastatic potential.

This figure presents representative Western blot (WB) analysis images that display the levels of CALML6 protein in human oral keratinocytes (HOK) and human oral cancer cell lines (SAT, HSC-4 and HSC-3) with varying metastatic potentials (*n*=1).

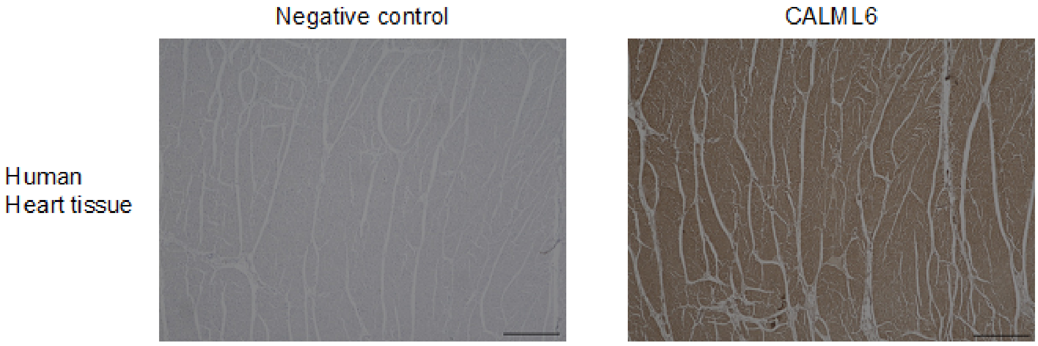

Supplemental Figure 8. Immunohistochemical staining of CALML6 in human heart tissue.

To validate the efficacy of the CALML6 antibody, we confirmed the expression of CALML6 protein in human heart tissue, serving as a positive control. Scale bars represent 500μm.

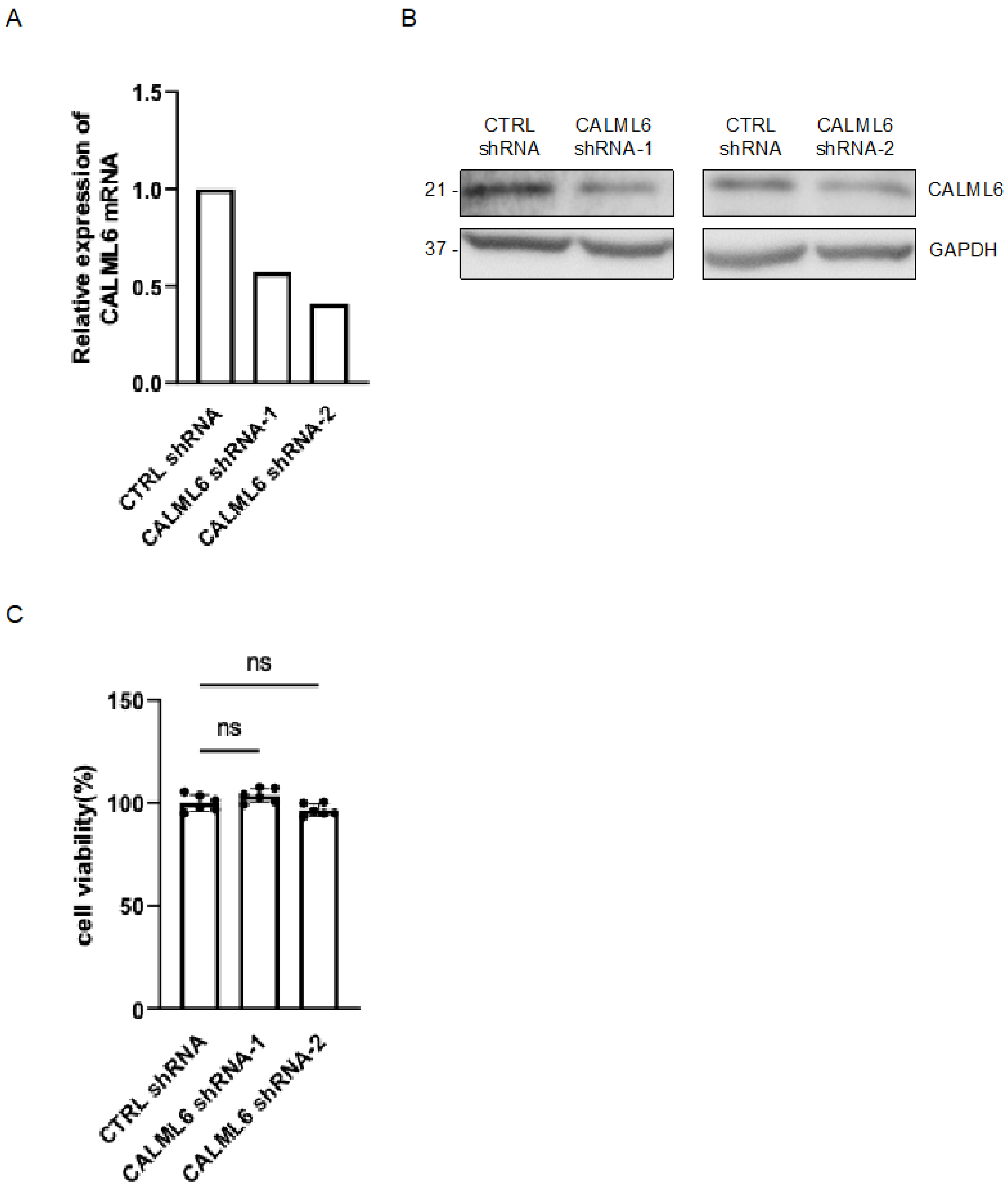

Supplemental Figure 9. Knockdown of CALML6 did not change the proliferation of OSCC cells.

A, Quantitative PCR analysis revealed that the mRNA levels of CALML6 in HSC-3 cells treated with either CALML6 shRNA-1 or CALML6 shRNA-2 lentivirus were significantly reduced

compared to those in HSC-3 cells treated with scramble shRNA lentivirus (n=1).

B, W.B. analyses revealed that the protein levels of CALML6 in HSC-3 cells treated with either CALML6 shRNA-1 or CALML6 shRNA-2 lentivirus were significantly reduced compared to those in HSC-3 cells treated with scramble shRNA lentivirus (n=1).

C, The XTT assay showed that knockdown of CALML6 did not change cell proliferation (unpaired t test; ns; not significant, n=6).

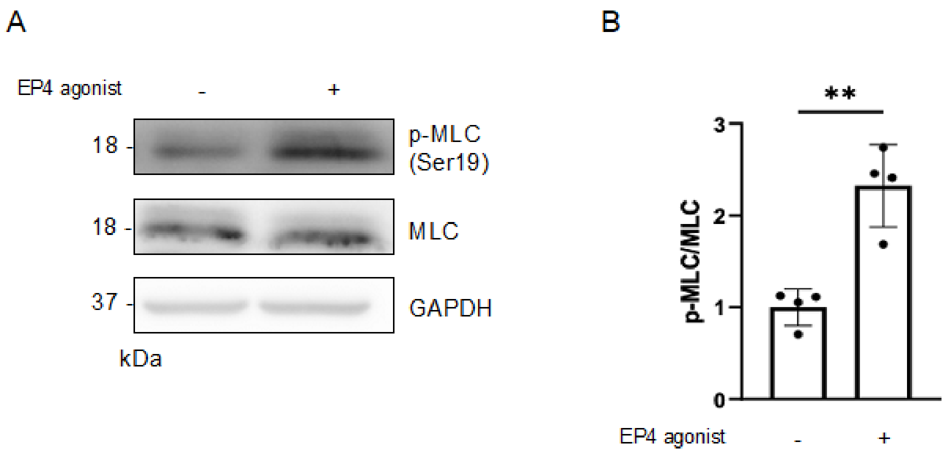

Supplemental Figure 10. Supplemental Figure 10. EP4 Agonist-Induced Phosphorylation of Myosin Light Chain (MLC) in OSCC Cell Lines.

A, This panel presents representative Western blot (WB) analysis images, showing the levels of phosphorylated MLC (p-MLC) in response to the EP4 agonist. The agonist induced phosphorylation of MLC at Ser19.

B, Densitometric analysis of the WB bands indicated a significant increase in MLC phosphorylation 30 minutes post-stimulation with EP4 agonist treatment (1 µM) (unpaired *t*-test; ***p*<0.01, *n*=4).

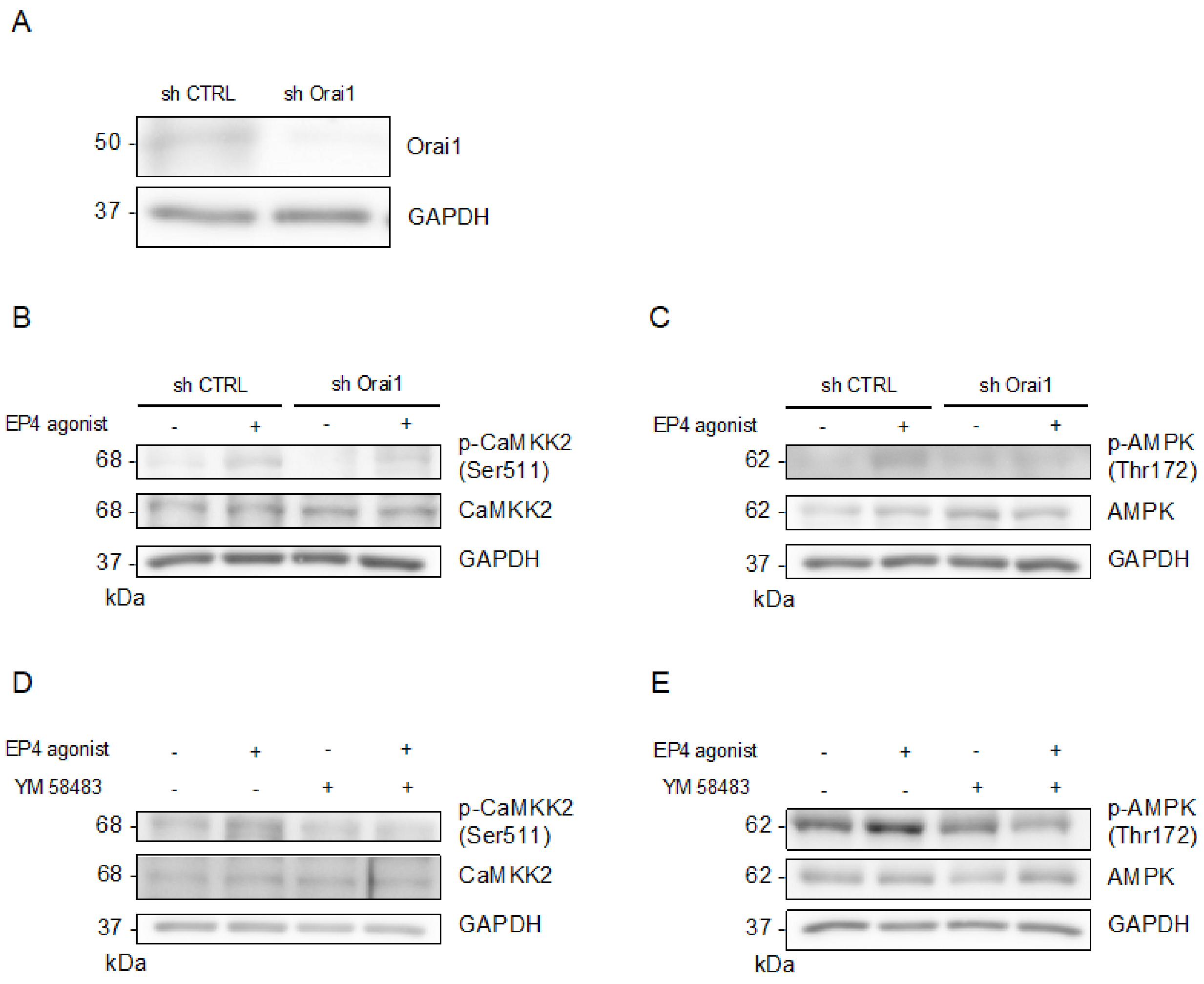

Supplemental Figure 11. Orai1 inhibited EP4 agonist-induced phosphorylation of CaMKK2 and AMPK.

A, W.B. analysis revealed that the protein level of Orai1 in HSC-3 cells treated with Orai1 shRNA lentivirus was significantly reduced compared to that in HSC-3 cells treated with scramble shRNA lentivirus (*n*=1).

B, Representative W.B. analysis images in HSC-3 cell lines are shown. Knockdown of Orai1 inhibited EP4 agonist-induced phosphorylation of CaMKK2 (*n*=1).

C, Representative W.B. analysis images in HSC-3 cell lines are shown. Knockdown of Orai1 inhibited EP4 agonist-induced phosphorylation of AMPK (*n*=1).

D, Representative W.B. analysis images in HSC-3 cell lines are shown. YM-58483 (a SOCE

inhibitor) inhibited EP4 agonist-induced phosphorylation of CaMKK2 (*n*=1). **e,** Representative W.B. analysis images in HSC-3 cell lines are shown. YM-58483 (a SOCE inhibitor) inhibited EP4 agonist-induced phosphorylation of AMPK (*n*=1).

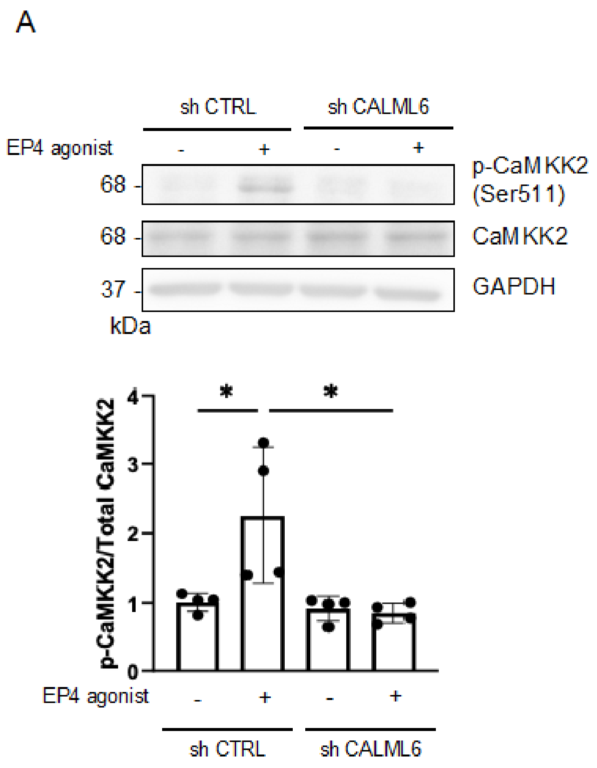

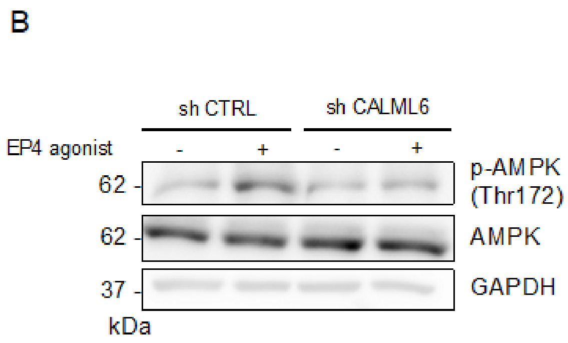

Supplemental Figure 12. The knockdown of CALML6 suppressed the EP4-indeuced CaMKK2 and AMPK phosphorylation in OSCC cells.

A, Representative W.B. analysis images of p-CaMKK2 in the presence or absence of the EP4 agonist are presented. Densitometric analyses of the W.B. analysis indicate a significant decrease in EP4 agonist-induced CaMKK2 phosphorylation when CALML6 shRNA lentivirus was applied, not scramble shRNA (One-way ANOVA, Tukey's multiple comparisons test; $*p < 0.05$). These findings are based on four independent experiments ($n = 4$).

B, Representative W.B. images of p-AMPK in the presence or absence of the EP4 agonist are provided. Densitometric analyses of the W.B. analysis indicate a significant decrease in EP4 agonist-induced AMPK phosphorylation when CALML6 shRNA lentivirus was applied, not scramble shRNA Data are representative of two independent experiments (n=2).

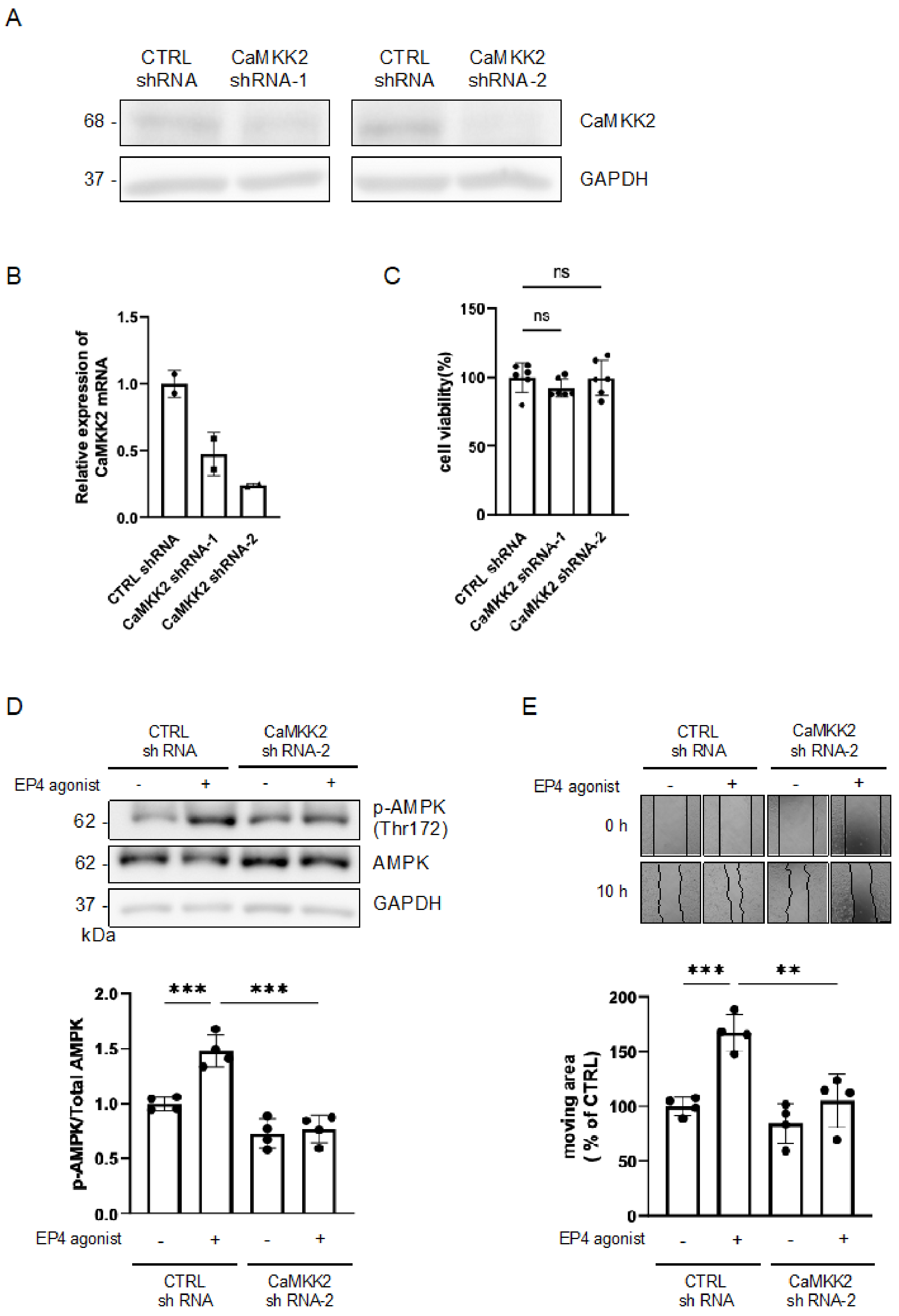
A
CTRL shRNA
CaMKK2 shRNA-1
CTRL shRNA
CaMKK2 shRNA-2
68 -
37 -
CaMKK2
GAPDH
B
Relative expression of CaMKK2 mRNA
1.5
1.0
0.5
0.0
CTRL shRNA
CaMKK2 shRNA-1
CaMKK2 shRNA-2
C
ns
ns
cell viability(%)
150
100
50
0
CTRL shRNA
CaMKK2 shRNA-1
CaMKK2 shRNA-2
D
CTRL sh RNA
CaMKK2 sh RNA-2
EP4 agonist
- + - +
62 -
62 -
37 -
kDa
p-AMPK (Thr172)
AMPK
GAPDH
***
***
p-AMPK/Total AMPK
2.0
1.5
1.0
0.5
0.0
EP4 agonist
- + - +
CTRL sh RNA
CaMKK2 sh RNA-2
E
CTRL sh RNA
CaMKK2 sh RNA-2
EP4 agonist
- + - +
0 h
10 h
***
**
moving area (% of CTRL)
200
150
100
50
0
EP4 agonist
- + - +
CTRL sh RNA
CaMKK2 sh RNA-2

Supplemental Figure 13. Knockdown of CaMKK2 suppressed EP4-induced AMPK phosphorylation and cell migration in OSCC cell lines.

A, W.B. analyses revealed that the protein levels of CaMKK2 in HSC-3 cells treated with either CaMKK2 shRNA-1 or CaMKK2 shRNA-2 lentivirus were significantly reduced compared to those in HSC-3 cells treated with scramble shRNA lentivirus (n=4).

B, Quantitative PCR analysis revealed that the mRNA levels of CaMKK2 in HSC-3 cells treated with either CaMKK2 shRNA-1 or CaMKK2 shRNA-2 lentivirus were significantly reduced compared to those in HSC-3 cells treated with scramble shRNA lentivirus (n=2).

C, The knockdown of CaMKK2 did not affect cell proliferation, as determined by the XTT assay (unpaired t test; ns; not significant, n=6).

D, Representative W.B. analysis images showing p-AMPK levels in the presence/absence of the EP4 agonist. Densitometric analysis of W.B. bands demonstrated that CaMKK2 shRNA-2 significantly diminished EP4-induced AMPK phosphorylation, similar to CaMKK2 shRNA-1 (right) (one-way ANOVA followed by Tukey's multiple comparisons test; ***p<0.001. Data are representative of n = 4 independent experiments).

E, Representative images of the scratch assay conducted in the presence or absence of the EP4 agonist are displayed (left). Densitometric analysis of pictures of scratch assay revealed that CaMKK2 shRNA-2 significantly reduced cell migration, similar to CaMKK2 shRNA-1 (right) (one-way ANOVA followed by Tukey's multiple comparisons test; **p<0.01, ***p<0.001. Data representative of n = 4 independent experiments). Scale bars, 200μm.

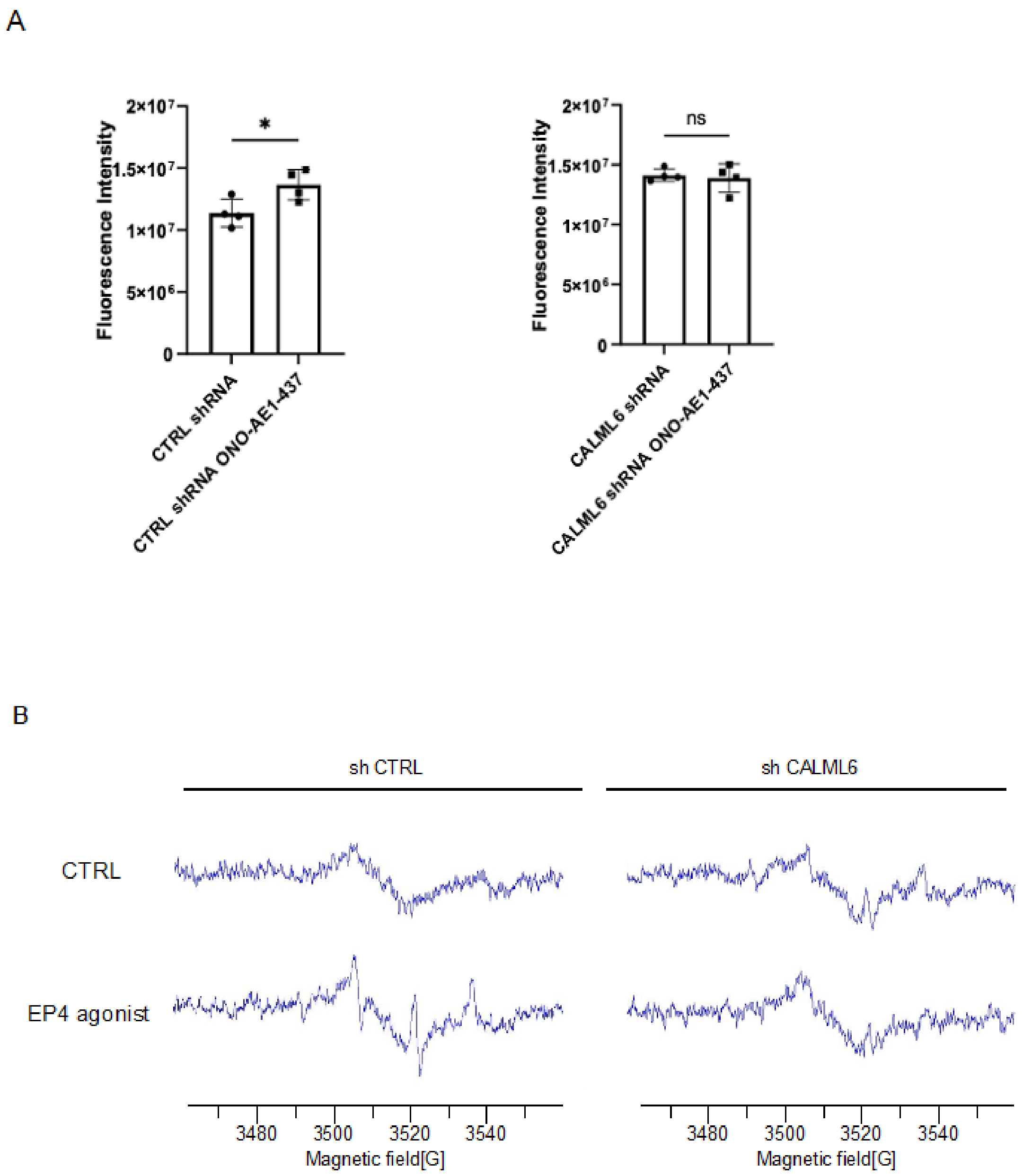

Supplemental Figure 14. Knockdown of CALML6 suppressed the EP4-indeuced ROS production in OSCC cells.

A, Knockdown of CALML6 reduced EP4-induced ROS production as measured by DCFH-DA assay (Unpaired *t*-test, ns; not significant; *n*=4, independent experiments).

B, Knockdown of CALML6 reduced EP4-induced ROS production as assessed by ESR (*n*=3, independent experiments).

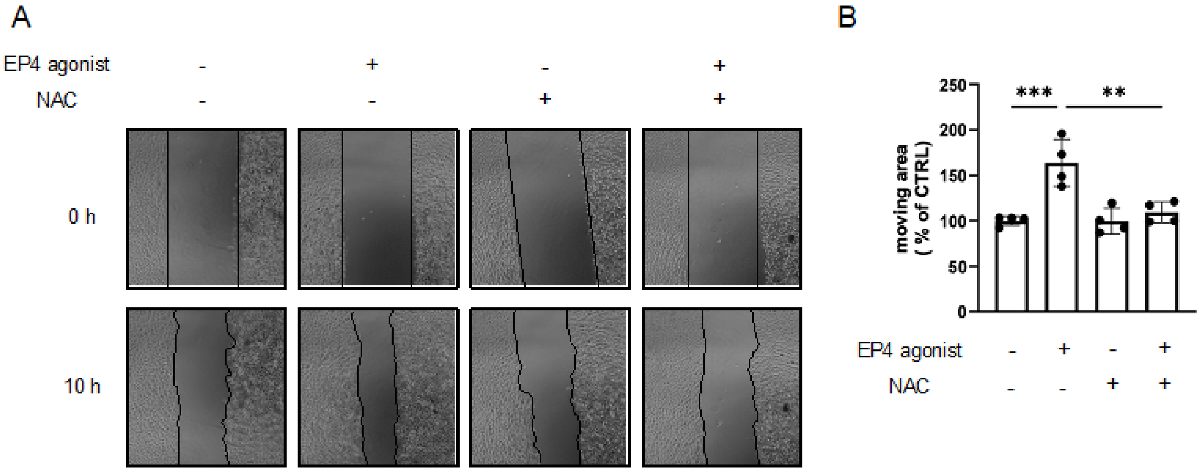

Supplemental Figure 15. Suppression of EP4-induced cell migration by an antioxidant in OSCC cell lines.

A, These panels show representative images from a scratch assay performed with OSCC cell lines treated with or without the EP4 agonist and N-Acetyl-L-cysteine (NAC).

B, Densitometric analysis of the scratch assay images indicates that NAC significantly reduced cell migration induced by EP4 agonist (one-way ANOVA followed by Tukey's multiple comparisons test; **p<0.01, ***p<0.001. Data representative of n = 4 independent experiments).

A

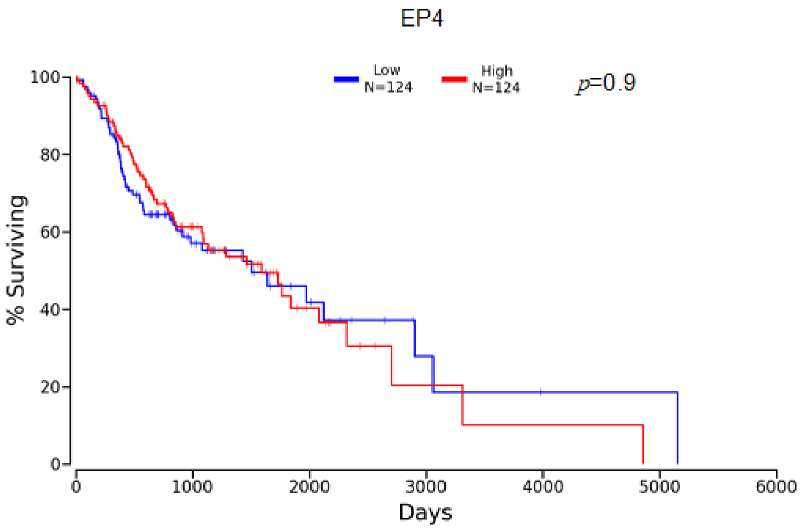

B

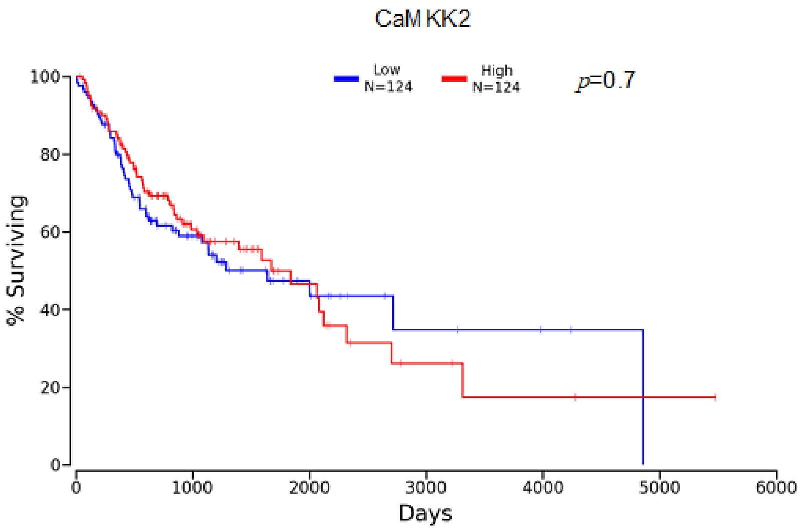

Supplemental Figure 16. Survival analysis of EP4 and CaMKK2 in head and neck squamous cell carcinoma (HNSCC) patients from The Cancer Genome Atlas (TCGA).

A, This panel presents an overall survival analysis of HNSCC patients, including those with oral squamous cell carcinoma (OSCC), based on EP4 mRNA expression. The analysis was performed using OncoLnc (http://www.oncolnc.org/).

B, The overall survival analysis for HNSCC patients, including OSCC patients, based on CaMKK2 mRNA expression, was similarly conducted using OncoLnc.

Supplemental Table 1. Short-heparin RNA transduction

Table S1 : Short-heparin RNA transduction		
Species	shRNA	Targeting sequences
Human	sh Control	CCTAAGGTTAAGTCGCCCTCG
Human	sh EP4-1	AGATGGTCATCTTACTCATTG
Human	sh EP4-2	ACTGAGGACTTTGCGAATATC
Human	sh CaMKK2-1	CCTGGCACTAATGGGAGTTTA
Human	sh CaMKK2-2	CGATGGTTTCCTGGCACTAAT
Human	sh CALML6-1	CCTGGCACTAATGGGAGTTTA
Human	sh CALML6-2	CGATGGTTTCCTGGCACTAAT
Human	sh Orail	GCAACGTGCACAATCTCAACT

Supplemental Table 2. Primer for RT-qPCR

Table S2 : Primer for RT-qPCR		
Species	Gene	sequence
Human	18S-F	GTAACCCGTTGAACCCCATT
Human	18S-R	CCATCCAATCGGTAGTAGCG
Human	CALML6-F	CCTGGCACTAATGGGAGTTT
Human	CALML6-R	TCCAGTCAATGTAGCCCTTG
Human	PGC1α-F	GTCACCACCCAAATCCTTAT
Human	PGC1α-R	ATCTACTGCCTGGAGACCTT
Human	mtDNA-F	CACCCAAGAACAGGGTTTGT
Human	mtDNA-R	TGGCCATGGGTATGTTGTTA
Human	TFAM-F	CCGAGGTGGTTTTCATCTGT
Human	TFAM-R	CGAGGTCTTTTTGGTTTTCC

Supplemental Figure 17. The uncropped blots

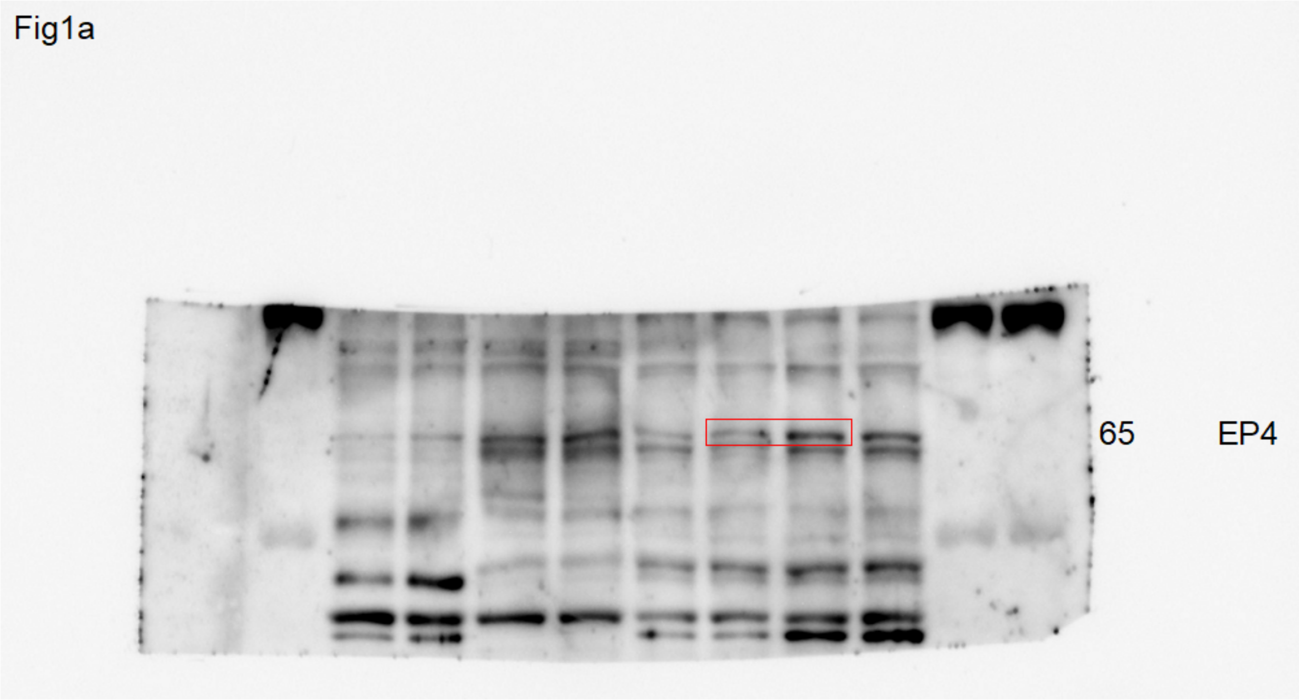

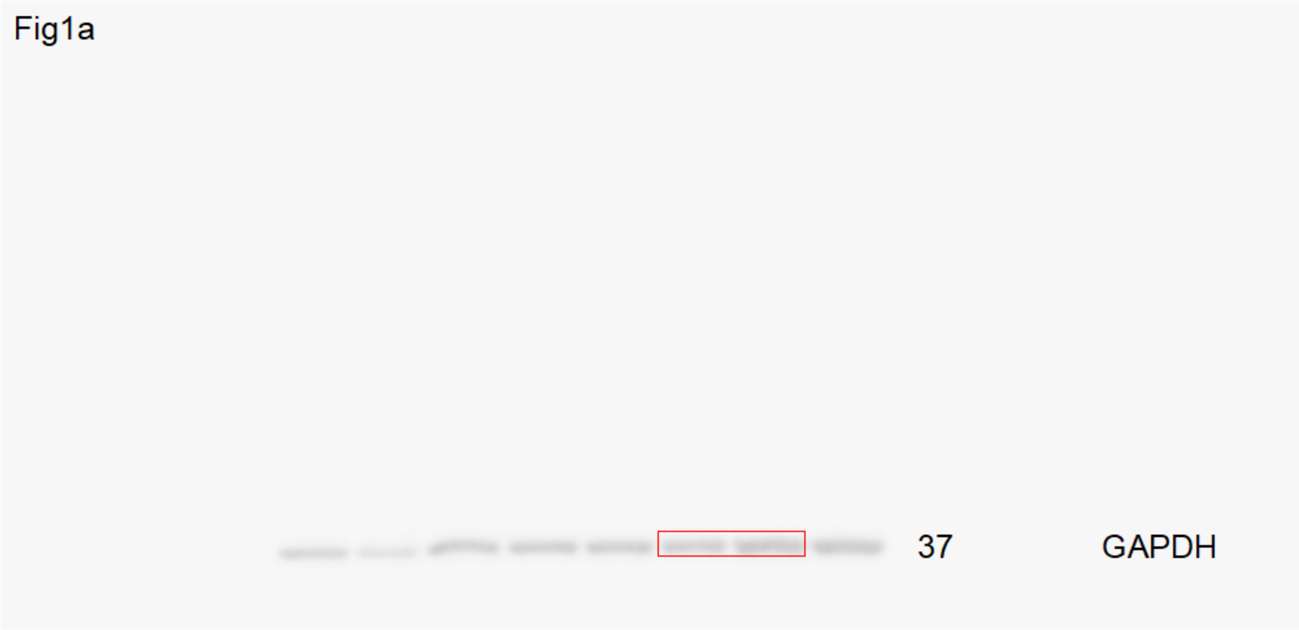

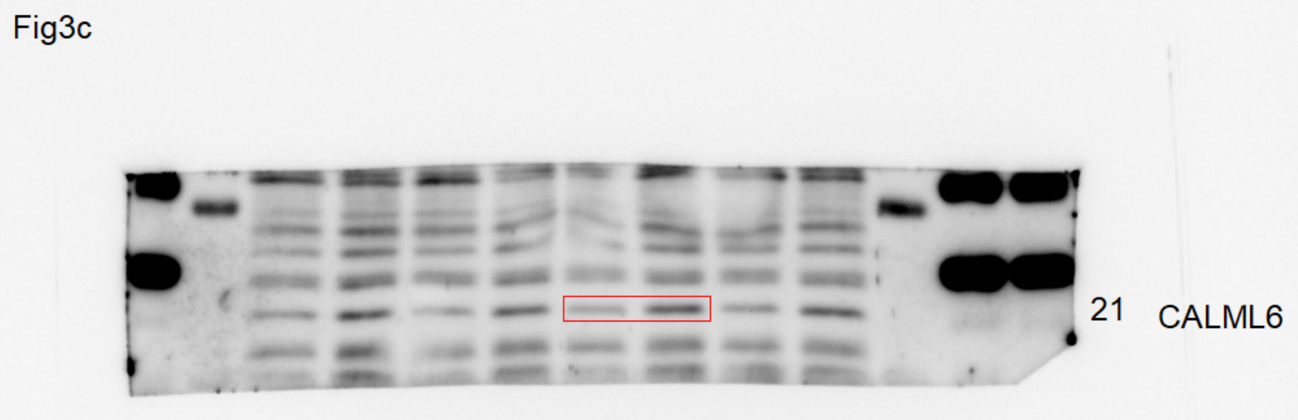
Fig3c
21
CALML6

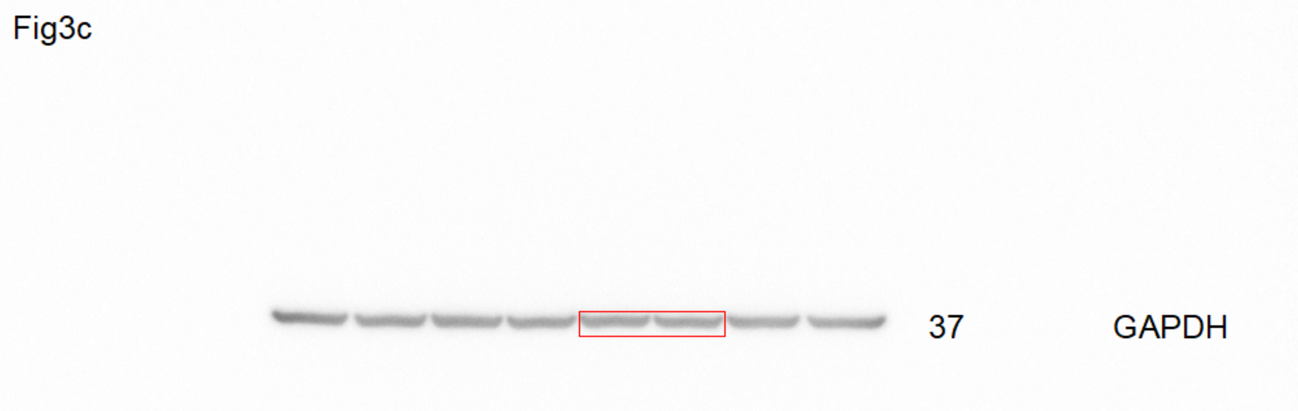
Fig3c
37
GAPDH

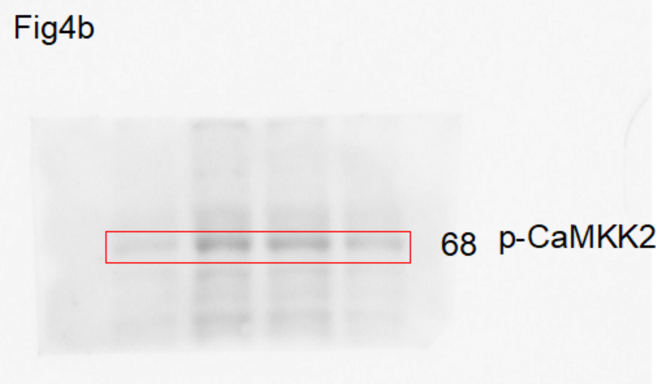
Fig4b
68
p-CaMKK2

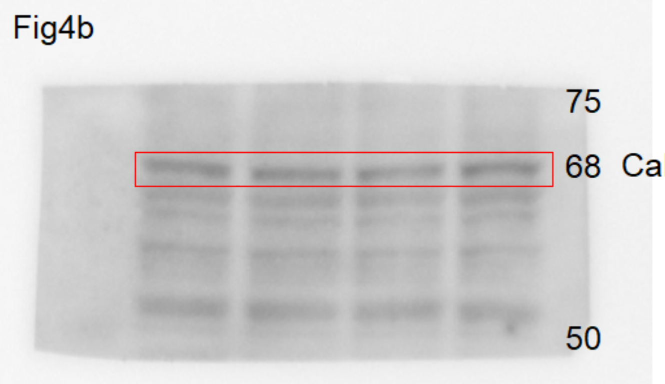
Fig4b
75
68
CaMKK2
50

Fig4b

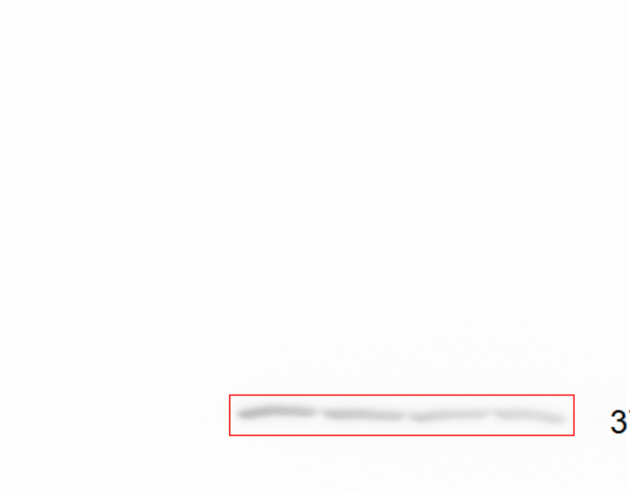

37 GAPDH

Fig4c

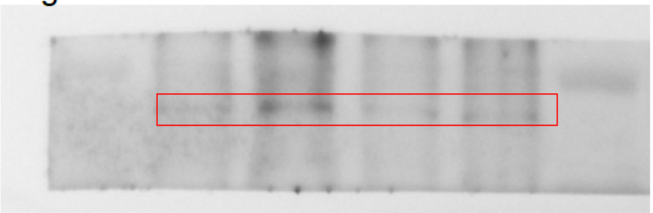

68 p-CaMKK2

Fig4c

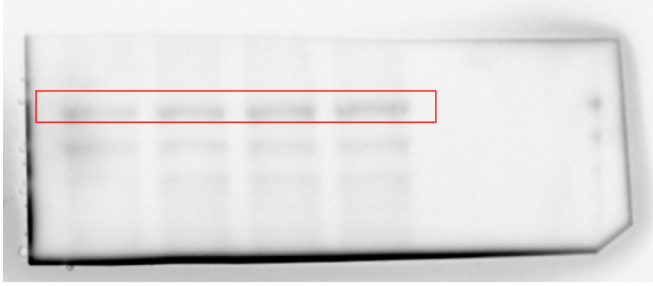

68 CaMKK2

Fig4c

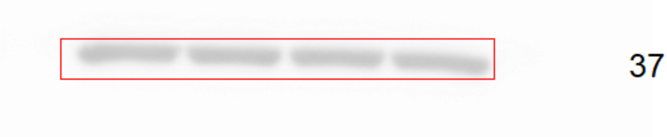

37 GAPDH

Fig4d

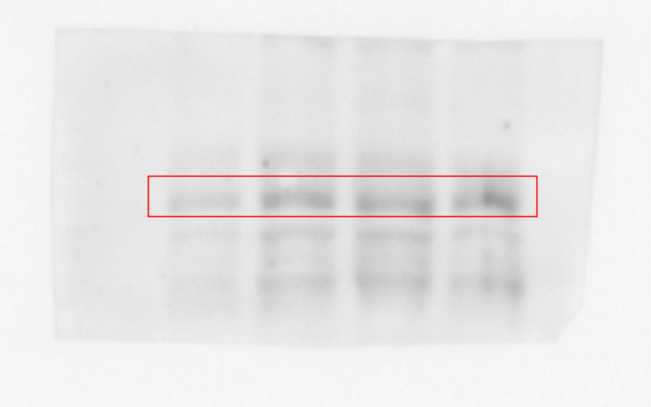

Fig4d

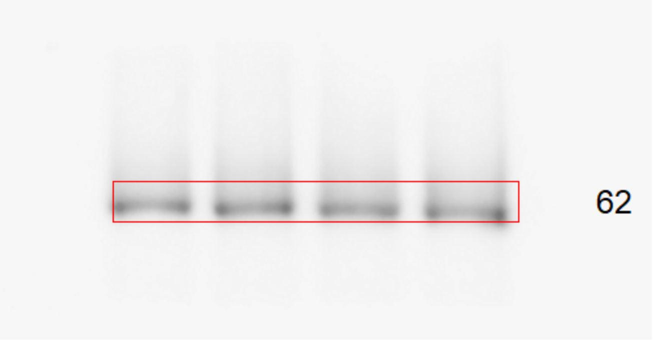

Fig4d

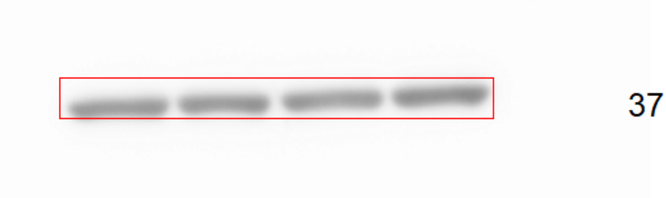

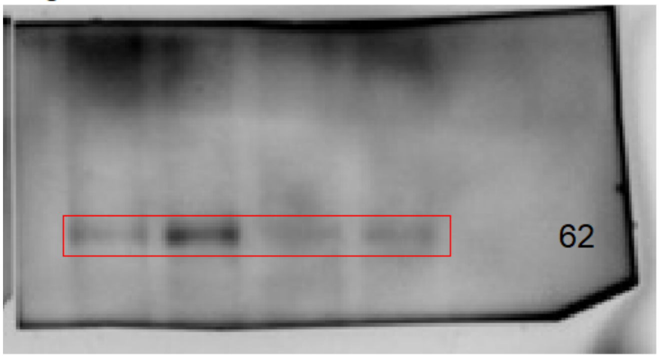

Fig5a

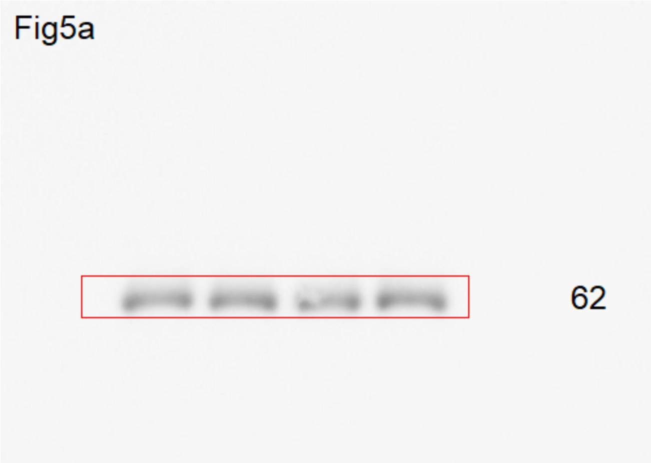

AMPK

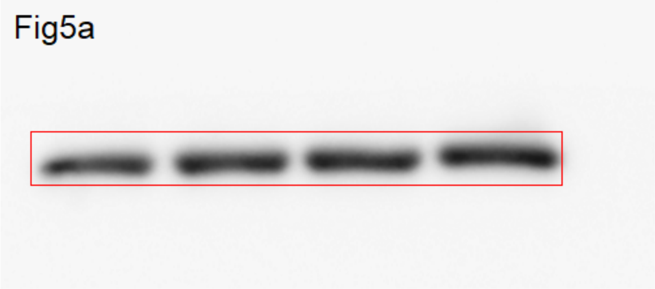

37 GAPDH

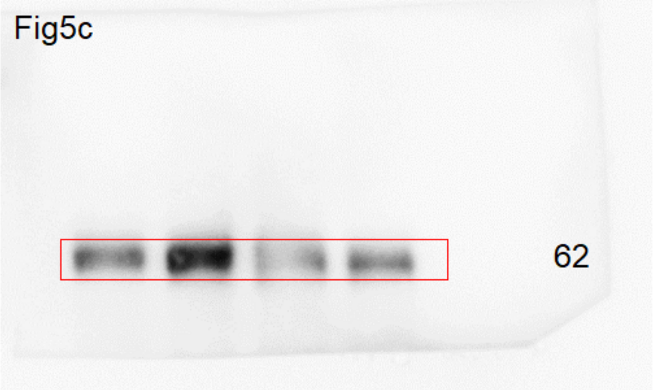

p-AMPK

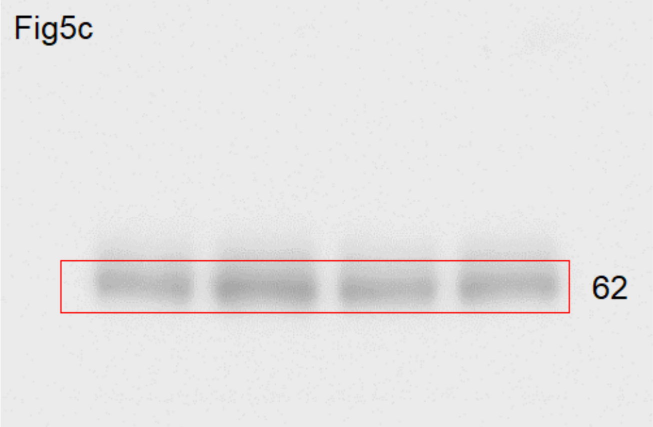

AMPK

Fig5c

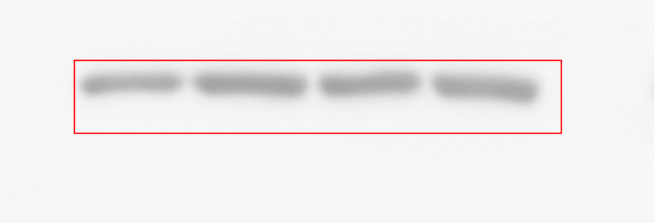

37 GAPDH

Fig6a

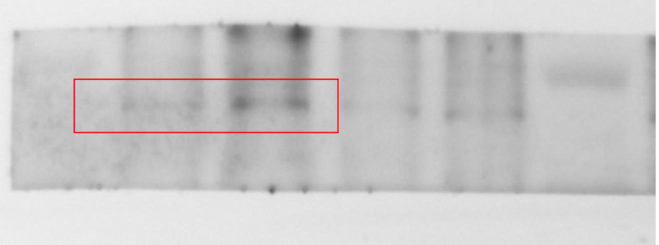

91 PGC1α

Fig6a

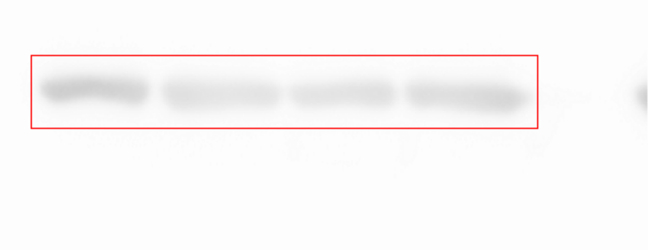

37 GAPDH

Supplementary Fig1A

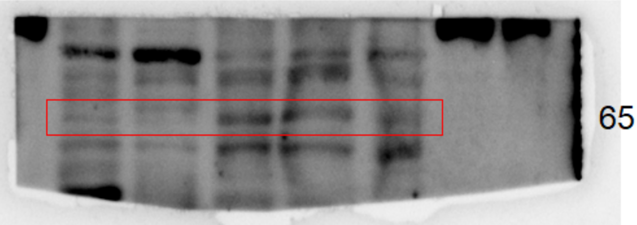

65 EP4

Supplementary Fig1A

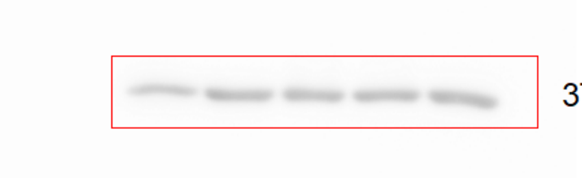

37 GAPDH

Supplementary Fig1B

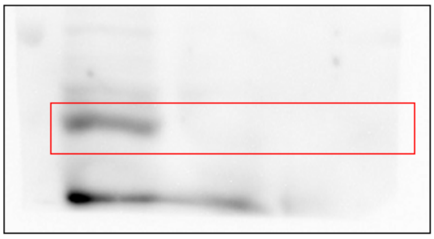

65 EP4

Supplementary Fig1B

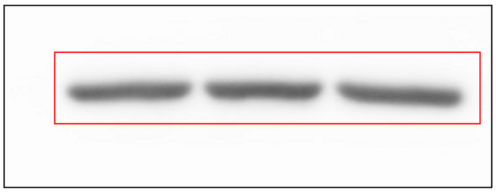

37 GAPDH

Supplementary Fig1C

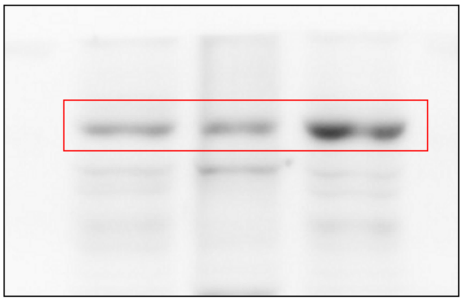

EP4

65

Supplementary Fig1C

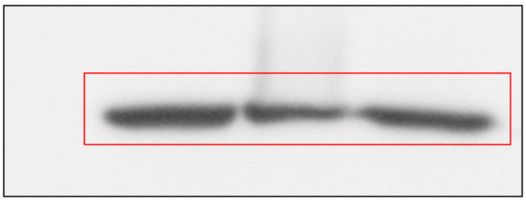

37 GAPDH

Supplementary Fig5B

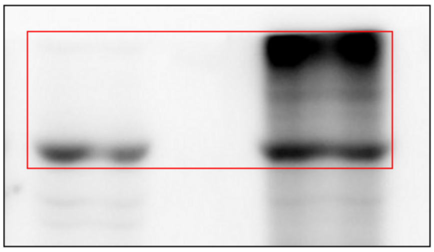

EP4

65

Supplementary Fig5B

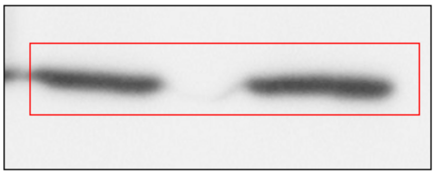

Supplementary Fig7

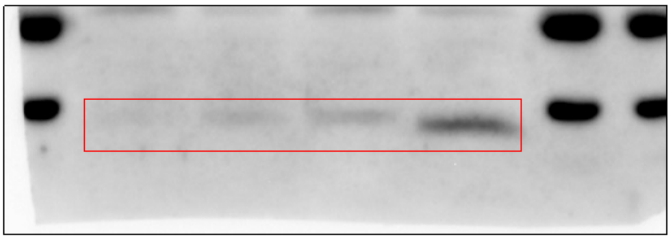

Supplementary Fig7

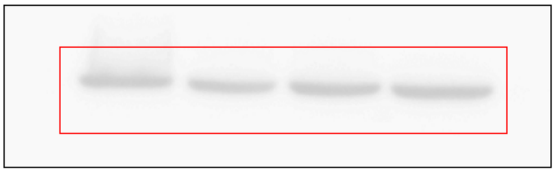

Supplementary Fig9

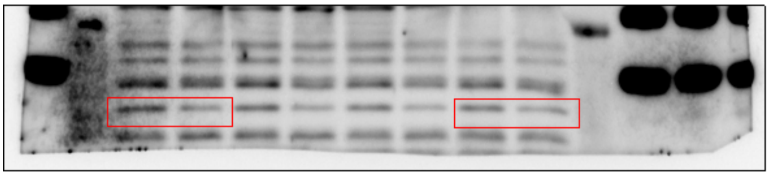

Supplementary Fig9

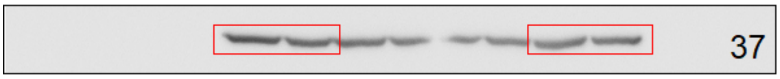

Supplementary Fig10

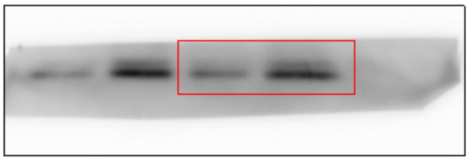

Supplementary Fig10

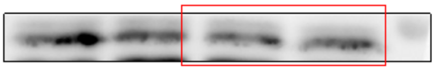

18 MLC

Supplementary Fiig10

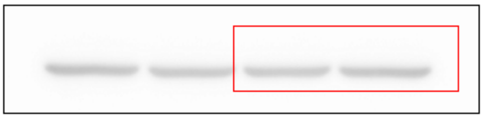

37 GAPDH

Supplementary Fig11a

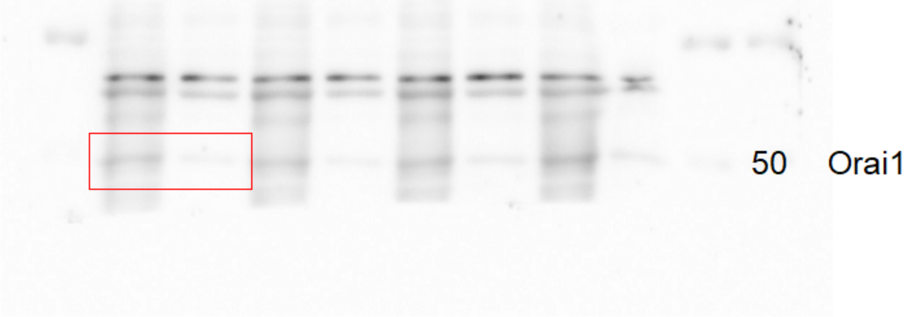

50 Orai1

Supplementary Fig11a

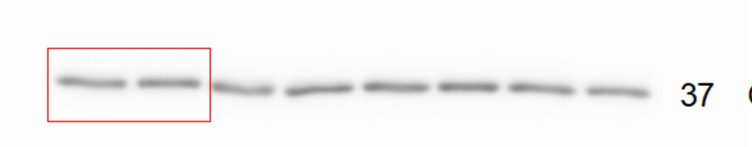

37 GAPDH

Supplementary Fig11b

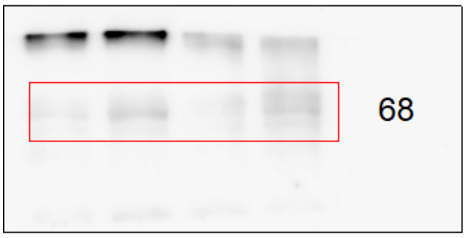

68 p-CaMKK2

Supplementary Fig11b

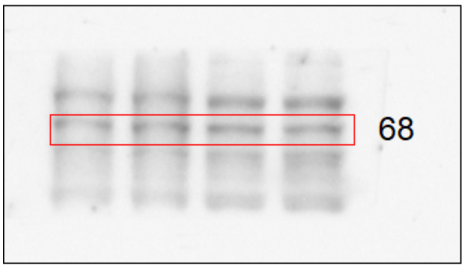

68 CaMKK2

Supplementary Fig11b

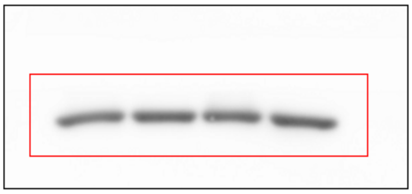

37 GAPDH

Supplementary Fig11c

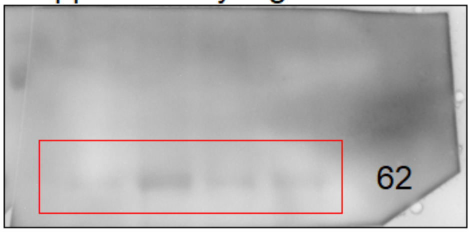

p-AMPK

Supplementary Fig11c

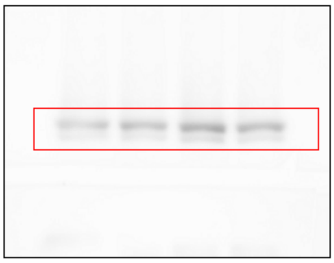

62 AMPK

Supplementary Fig11c

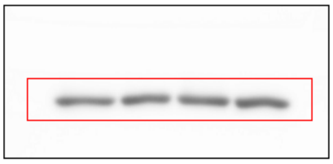

37 GAPDH

Supplementary Fig11d

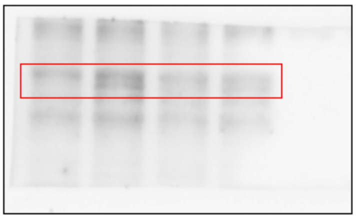

68 p-CaMKK2

Supplementary Fig11d

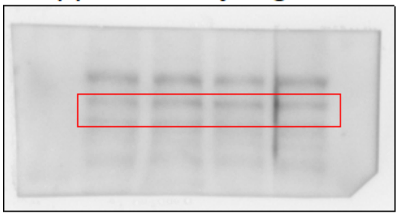

68 CaMKK2

Supplementary Fig11d

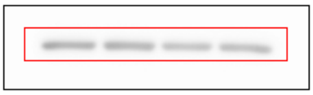

37 GAPDH

Supplementary Fig11e

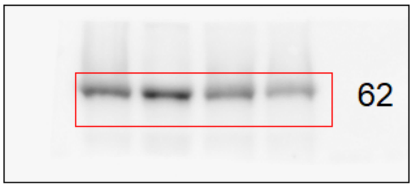

Supplementary Fig11e

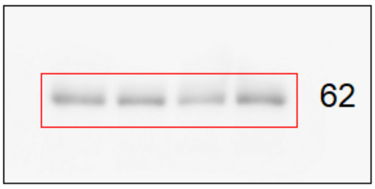

AMPK

Supplementary Fig11e

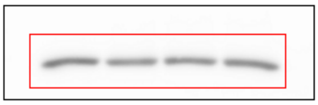

37 GAPDH

Supplementary Fig12a

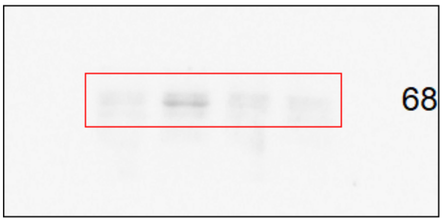

68 p-CaMKK2

Supplementary Fig12a

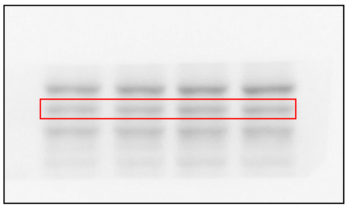

68 CaMKK2

Supplementary Fig12a

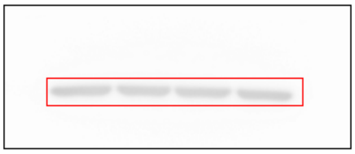

37 GAPDH

Supplementary Fig12b

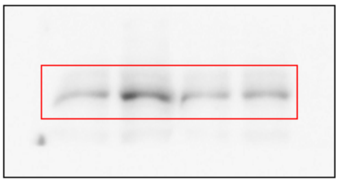

62 p-AMPK

Supplementary Fig12b

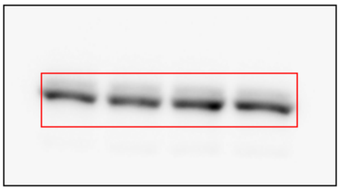

62 AMPK

Supplementary Fig12b

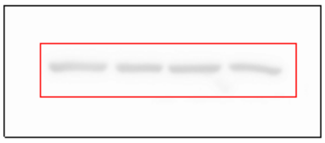

37 GAPDH

Supplementary Fig13a

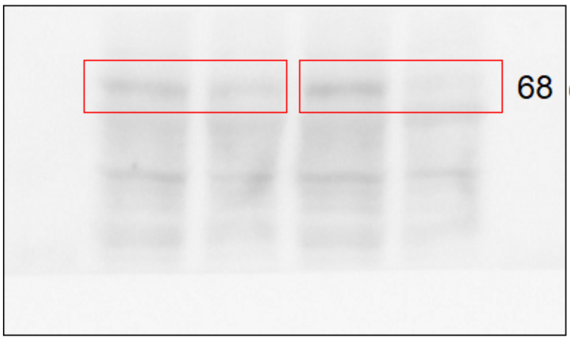

68 CaMKK2

Supplementary Fig13a

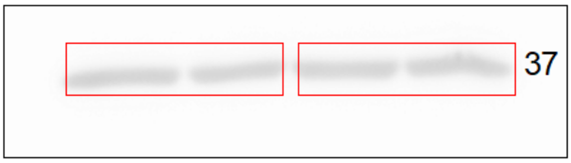

37 GAPDH

Supplementary Fig13d

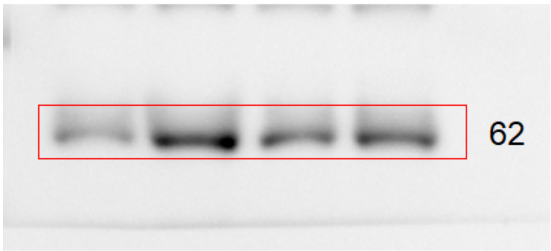

62 p-AMPK

Supplementary Fig13d

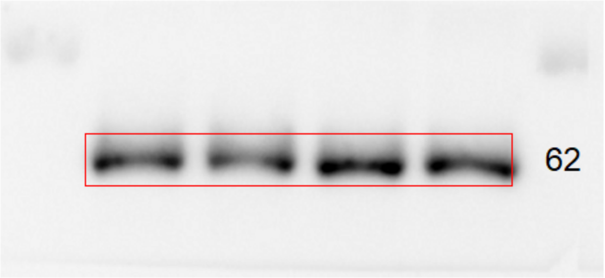

62 AMPK

Supplementary Fig13d

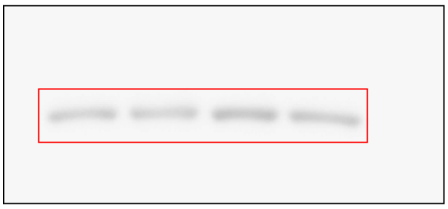

37 GAPDH
