# Supplementary material for: EP4-induced mitochondrial localization and cell migration mediated by CALML6 in human oral squamous cell carcinoma
Source: Commun Biol. 2024 May 14;7:567. doi: 10.1038/s42003-024-06231-4 (PMC11093972; doi:10.1038/s42003-024-06231-4)
Supplement: Supplementary file 3 — Description of Additional Supplementary Files [file 42003_2024_6231_MOESM3_ESM.pdf]

Description of Additional Supplementary Files

File name: Supplementary Data 1
Description: The source data behind the graphs in the paper can be found in Supplementary Data 1.

File name: Supplementary Movie 1
Description: Time-lapse images of HGnF and HSC-3 migration studies are shown.

File name: Supplementary Movie 2
Description: Time-lapse images of migration studies of CTRL and EP4 overexpressing cells are shown.

File name: Supplementary movie 3
Description: Time-lapse images of migration studies of shCTRL and sh CALML6 cells with or without EP4 agonist are shown.

File name: Supplementary movie 4
Description: Time-lapse images of migration studies of EP4 agonist-stimulated and STO-609-stimulated in HSC-3 cells are shown.

File name: Supplementary movie 5
Description: Time-lapse images of migration studies of shCTRL and sh CaMKK2 cells with or without EP4 agonist are shown.

File name: Supplementary movie 6
Description: Time-lapse images of migration studies of EP4 agonist-stimulated and NAC-stimulated in HSC-3 cells are shown.

File name: Supplementary movie 7
Description: Time-lapse images of cell tracking assay in HSC-3 with or without EP4 agonist are shown.
