# Supplementary material for: EP4-induced mitochondrial localization and cell migration mediated by CALML6 in human oral squamous cell carcinoma
Source: Commun Biol. 2024 May 14;7:567. doi: 10.1038/s42003-024-06231-4 (PMC11093972; doi:10.1038/s42003-024-06231-4)
Supplement: Supplementary file 12 — Reporting Summary [file 42003_2024_6231_MOESM12_ESM.pdf]

Corresponding author(s): Masanari Umemura
Yoshihiro Ishikawa

Last updated by author(s): Apr 2, 2024

Reporting Summary

Nature Portfolio wishes to improve the reproducibility of the work that we publish. This form provides structure for consistency and transparency in reporting. For further information on Nature Portfolio policies, see our Editorial Policies and the Editorial Policy Checklist.

Statistics

For all statistical analyses, confirm that the following items are present in the figure legend, table legend, main text, or Methods section.

n/a	Confirmed	
☐	☒	The exact sample size (n) for each experimental group/condition, given as a discrete number and unit of measurement
☐	☒	A statement on whether measurements were taken from distinct samples or whether the same sample was measured repeatedly
☐	☒	The statistical test(s) used AND whether they are one- or two-sided<br>*Only common tests should be described solely by name; describe more complex techniques in the Methods section.*
☒	☐	A description of all covariates tested
☒	☐	A description of any assumptions or corrections, such as tests of normality and adjustment for multiple comparisons
☐	☒	A full description of the statistical parameters including central tendency (e.g. means) or other basic estimates (e.g. regression coefficient) AND variation (e.g. standard deviation) or associated estimates of uncertainty (e.g. confidence intervals)
☐	☒	For null hypothesis testing, the test statistic (e.g. F, t, r) with confidence intervals, effect sizes, degrees of freedom and P value noted<br>*Give P values as exact values whenever suitable.*
☒	☐	For Bayesian analysis, information on the choice of priors and Markov chain Monte Carlo settings
☒	☐	For hierarchical and complex designs, identification of the appropriate level for tests and full reporting of outcomes
☒	☐	Estimates of effect sizes (e.g. Cohen's d, Pearson's r), indicating how they were calculated

Our web collection on statistics for biologists contains articles on many of the points above.

Software and code

Policy information about availability of computer code

Data collection	The chemiluminescent signal of Western blotting was acquired by LuminoGraph II imaging system.<br>Scratch wound healing assay and cell tracking migration assay were collected using ECLIPSE Ti.<br>Invasion assay, immunocytochemistry, immunohistochemistry were using BZ-X800.<br>Fluorescence imaging, ATP assay, ROS assay were using Nivo.<br>The pictures of animal experiments were using IVIS.
Data analysis	Scratch wound healing assay was analyzed using NIS-Elements AR.<br>Western blotting images were analyzed using CS Analyzer 4.<br>Statistical analyses were performed using GraphPad Prism 9.

For manuscripts utilizing custom algorithms or software that are central to the research but not yet described in published literature, software must be made available to editors and reviewers. We strongly encourage code deposition in a community repository (e.g. GitHub). See the Nature Portfolio guidelines for submitting code & software for further information.

Data

Policy information about availability of data

All manuscripts must include a data availability statement. This statement should provide the following information, where applicable:

- Accession codes, unique identifiers, or web links for publicly available datasets
- A description of any restrictions on data availability
- For clinical datasets or third party data, please ensure that the statement adheres to our policy

All data of this study are available from the corresponding author upon reasonable request. All source data , uncropped and unedited blot/gel images are availablle in Dataset files

Research involving human participants, their data, or biological material

Policy information about studies with human participants or human data. See also policy information about sex, gender (identity/presentation), and sexual orientation and race, ethnicity and racism.

Reporting on sex and gender	N/A
Reporting on race, ethnicity, or other socially relevant groupings	N/A
Population characteristics	N/A
Recruitment	N/A
Ethics oversight	N/A

Note that full information on the approval of the study protocol must also be provided in the manuscript.

Field-specific reporting

Please select the one below that is the best fit for your research. If you are not sure, read the appropriate sections before making your selection.

☒ Life sciences ☐ Behavioural & social sciences ☐ Ecological, evolutionary & environmental sciences

For a reference copy of the document with all sections, see nature.com/documents/nr-reporting-summary-flat.pdf

Life sciences study design

All studies must disclose on these points even when the disclosure is negative.

Sample size	Sample size was chosen based on our prior studies using the same types of assays. All experiments were performed at least three times or with at least three biological replicates.
Data exclusions	No data were excluded in this study.
Replication	The data of in vitro assay was performed with at least three independent experiments. The data from animal experiments was performed with biological replicates.
Randomization	Mice were randomized to treatment groups.
Blinding	The experiments were not blinded as the experimental observations would be irrespective of blinding.

Reporting for specific materials, systems and methods

We require information from authors about some types of materials, experimental systems and methods used in many studies. Here, indicate whether each material, system or method listed is relevant to your study. If you are not sure if a list item applies to your research, read the appropriate section before selecting a response.

Materials & experimental systems

n/a	Involved in the study	
☐	☒	Antibodies
☐	☒	Eukaryotic cell lines
☒	☐	Palaeontology and archaeology
☐	☒	Animals and other organisms
☒	☐	Clinical data
☒	☐	Dual use research of concern
☒	☐	Plants

Methods

n/a	Involved in the study	
☒	☐	ChIP-seq
☒	☐	Flow cytometry
☒	☐	MRI-based neuroimaging

Antibodies

Antibodies used

CaMKK2, #16810, Cell signaling technology, 1:1000 (WB)
p-CaMKK2 (Ser511), #12818, Cell signaling technology, 1:1000 (WB)
AMPK, #5832, Cell signaling technology, 1:1000 (WB)
p-AMPK (Thr172), #2535, Cell signaling technology, 1:1000 (WB)
GAPDH, sc-32233, Santa Cruz Biotechnology, 1:1000 (WB)
EP4, 101775, Cayman Chemical, 1:1000 (WB) , 1:100 (IF)
CALML6, 22274-1-AP, Proteintech, 1:1000 (WB), 1:50 (IHC)
PGC1-α, ab191838, abcam, 1:1000 (WB)
MLC, #3672, Cell signaling technology, 1:1000 (WB)
p-MLC (Ser19), #3671, Cell signaling technology, 1:1000 (WB)

Validation

All commercially available antibodies were validated by the suppliers. Details can be referred on the manufactures' websites.

Eukaryotic cell lines

Policy information about cell lines and Sex and Gender in Research

Cell line source(s)

The cell lines sources have been described in detail in Materials and Methods.

Authentication

All cell lines were authenticated.

Mycoplasma contamination

All cell lines were negative.

Commonly misidentified lines (See ICLAC register)

No commonly misidentified cell lines were used.

Animals and other research organisms

Policy information about studies involving animals; ARRIVE guidelines recommended for reporting animal research, and Sex and Gender in Research

Laboratory animals

Four-to five-week-old BALB/c Slc-nu/nu female mice were used for animal experiments.

Wild animals

No wild animals were used in this study.

Reporting on sex

Female mice were used in our study.

Field-collected samples

No field-collected samples were used in this study.

Ethics oversight

All animal experiments were approved by the Animal Care and Use Committee at Yokohama City University School of Medicine (F-A-22-044).

Note that full information on the approval of the study protocol must also be provided in the manuscript.

Plants

Seed stocks	N/A
Novel plant genotypes	N/A
Authentication	N/A
